# Supplementary material for: Uncovering complementary information sharing in spider monkey collective foraging using higher-order spatial networks
Source: Npj Complex. 2026 Jan 9;3(1):1. doi: 10.1038/s44260-025-00060-0 (PMC12789017; doi:10.1038/s44260-025-00060-0)
Supplement: Supplementary file 1 — Supplementary Information [file 44260_2025_60_MOESM1_ESM.pdf]

Supplementary information for Uncovering complementary information sharing in spider monkey collective foraging using higher-order spatial networks

Gabriel Ramos-Fernandez, Ross S. Walker, Matthew J. Silk, Denis Boyer and Sandra E. Smith Aguilar

This document contains further methodological detail on the study subjects and the derivation of the optimal relative spatial overlap in section 1, while section 2 contains additional results on: 1) the correlation between the filtration complementarity index and the seasons and the variation in fruit abundance; and 2) the maximal simplicial centrality analysis and the individual composition of simplices.

1 Methods

1.1 Further data details

ID	Sex	Birth date	Age Jan2012	Age Jan2017	2012 dry		2012 wet		2013 dry		2013 wet		2014 dry		2014 wet		2015 dry		2015 wet		2016 dry		2016 wet		2017 dry		2017 wet	
					Samples	Days	Samples	Days	Samples	Days	Samples	Days	Samples	Days	Samples	Days	Samples	Days	Samples	Days	Samples	Days	Samples	Days	Samples	Days	Samples	Days
AE	F	2/28/07	4.9	10							137	27	206	37	180	48							180	39	175	31		
AI	M	7/22/01	10.6	NA													101	18										
AM	F	5/23/01	10.8	NA			133	24	160	28	227	36	159	26	242	51												
BE	M	7/1/94	17.8	NA					104	20	121	25																
BH	F	9/4/06	5.4	NA							292	41																
BO	M	7/22/01	10.6	NA					136	32	153	31	121	22	174	35												
CH	F	1/1/83	29.4	34.5	267	40	275	49	530	62	520	70	487	59	284	56	311	60	264	47	359	56	494	71	377	53	370	47
EG	M	1/16/04	8.1	13.2	144	19	112	28	186	29	370	51	355	55	303	54	481	51	407	56	503	58	302	59	267	51	179	27
EL	F	9/4/08	3.4	8.4																			166	29	178	37		
FL	F	1/1/86	26.4	31.5	286	41	242	48	352	51	422	59	252	39	364	65	290	55	117	32	175	37	300	52	242	39	231	29
HI	F	4/20/04	7.8	12.9			105	25			128	30	173	27	185	44	132	27			158	31	397	57	277	51		
JA	F	7/22/98	13.6	18.7	321	38	344	58	395	53	401	57	393	58	399	69	290	55	135	38	188	43	357	64	343	57	224	31
JN	M	12/1/03	8.2	13.3	127	24			243	34	278	45	306	43	368	64	699	64	529	57	593	60	381	65	229	48	216	29
KL	F	9/10/96	15.5	20.6	193	30	129	30	166	25	318	41	232	43	337	55	183	35					207	43	158	34	121	17
KO	M	4/6/10	1.8	6.8																			249	52	213	47	154	26
LO	F	11/11/01	10.3	15.4	263	36	326	50	541	62	549	67	498	62	334	57	268	55	239	44	329	51	471	72	315	49	200	29
ME	F	9/28/08	3.3	NA											373	65	177	33										
ML	F	8/14/05	6.5	11.6											185	49					140	34	281	51	252	49		
MM	F	3/20/09	2.8	7.9																			203	45	176	38	135	23
MS	M	7/15/07	4.5	9.6					358	44	428	57	374	53	364	57	659	63	533	63	605	61	400	62	213	46	165	21
PC	F	8/16/02	9.5	14.6									158	29	232	46					113	28	272	53	216	45		
SK	F	5/17/07	4.7	NA							104	27																
TG	F	8/16/02	9.5	14.6							108	17	181	40	151	33							250	43	180	37		
TL	F	8/6/01	10.6	15.6	127	20	162	37	265	42	324	47	350	49	336	52	508	47	448	56	595	59	323	56	260	55	168	22
VA	F	12/11/09	2.1	7.2															239	43	322	53	450	67	290	49	186	28
VE	F	1/1/84	28.4	33.5	328	38	210	46	469	60	444	65	324	51	374	64	309	61	200	37	259	45	524	81	414	60	329	45
VI	F	12/23/07	4.1	NA					343	42																		
WB	M	12/11/10	1.1	6.1																			330	48	168	43		

Table SI1: sample size, in terms of the number of scan samples and days of observation, in each season for each individual spider monkey included in the study. Also shown are the sex and date of birth, as well as the age of each individual in the first and last years of this study.

1.2 Optimal space sharing and subgroup size

In this subsection we pose and prove the result that the optimal relative spatial overlap between all individuals (in a subgroup of size n) is exactly

$$w_n^* = \frac{1}{n+1} \tag{1}$$

In the main paper this result was used to determine how redundant were the spatial interactions between core ranges observed from the data when taking into account subgroup size. This section

first constructs a suitable objective function which describes the total level of information transfer happening in a subgroup of n individuals under a particular spatial overlap structure. Then, assuming that each individual has equal foraging ability, we prove global optimality of a particular spatial structure which corresponds to the result (1).

1.2.1 Construction of an objective function

We want to find the 'area overlap' between home ranges which maximises the information transfer between n individuals. For this, we need to construct an objective function (the *objective function*) and a suitable set of constraints on the optimisation problem.

The union of the core ranges of all individuals are partitioned into areas known uniquely by distinct subsets of individuals. The areas which are known by multiple individuals will be the variables of the objective function. Throughout, we make the following assumptions:

1. Individuals utilise the area in their core ranges uniformly.
2. core ranges are composed of cells, representing, for example, points of knowledge.
3. Movements of different individuals are independent.
4. Rate of information transfer for a given grouping of individuals is given by the number of cells known by the group known by at least one member of the group and not known by at least one member, multiplied by the probability of the interaction occurring.

Suppose we have n-many individuals, enumerated from 1 to n. By Assumption 2, the i-th individual has a core range consisting of N_i many cells for each $i = 1, \ldots, n$. Denote by $[n]$ the set $\{1, \ldots, n\}$. Denote the power set of $[n]$ by $\mathcal{P}([n])$. Then, the set of all multi-individual interactions, denoted $\mathcal{X}$, will be $\mathcal{P}([n])$ excluding singletons and the empty set $\emptyset$. The size of this set, $\mathcal{N} = |\mathcal{X}|$, will then be

$$\mathcal{N} = \underbrace{2^n}_{\text{size of } \mathcal{P}([n])} - \underbrace{n}_{\text{number of singletons in } \mathcal{P}([n])} - \underbrace{1}_{\text{the empty set } \emptyset}$$

(which implies that the optimisation problem will be $2^n - n - 1$ dimensional). Each element of χ will be of the form $\{i_1, ..., i_k\}$ where each $i_l \in [n]$. We can arrange the entries of each of these elements so $i_1, ..., i_k$ is an increasing sequence. Then we can then place an ordering upon $\mathcal{X}$ by arranging the elements in ascending cardinality (so interactions between fewer individuals come before interactions between more individuals) and then order elements of the *same* cardinality lexicographically. Some examples: $\{1, 2\}$ will be the first element in the ordering, $\{1, 2, 3\}$ will be the first element of size 3 and $[n]$ (the set of all individuals) will be the final element. This ordering allows us to enumerate the overlapping areas according to their position in the ordering of $\mathcal{X}$. We represent the enumeration with the (bijective) mapping $I\colon \mathcal{X} \to [\mathcal{N}]$, which matches a group of individuals with its index in the ordering. Equivalently, for a given $c \in [\mathcal{N}]$, $I^{-1}(c)$ gives the c-th group of individuals in the ordering of $\mathcal{X}$. With this construction we can list all of our area overlaps (the variables of our objective function) as $(O_1, \ldots, O_{\mathcal{N}})$. Note that this particular choice of ordering was chosen to maintain interpretability of results and ensure easy compatibility with optimisation software, although the choice is mathematically arbitrary.

We account for the fact that any grouping of individuals can interact in both the areas that they share uniquely *and* in the areas shared by them and additional individuals. For example, individuals 1 and 2 can meet in the area shared by individuals 1, 2 and 3 without the presence of individual 3. This motivates the following definition. Let $S\colon [\mathcal{N}] \to \mathcal{P}([\mathcal{N}])$ be a mapping such that $S(c)$ is the collection of indices of areas known by the individuals $I^{-1}(c)$. We can additionally write

$$S(c) = \left\{ i \in [\mathcal{N}] : I^{-1}(c) \subseteq I^{-1}(i) \right\}.$$

For example, in the case $n = 3$, we have $\mathcal{X} = \{\{1, 2\}, \{1, 3\}, \{2, 3\}, \{1, 2, 3\}\}$, with ordering map given by $I(\{1, 2\}) = 1$, $I(\{1, 3\}) = 2$, $I(\{2, 3\}) = 3$ and $I(\{1, 2, 3\}) = 4$. The corresponding S mapping is $S(1) = \{1, 4\}$, $S(2) = \{2, 4\}$, $S(3) = \{3, 4\}$, $S(4) = \{4\}$. With these constructions we can finally write that, for a given group of individuals, with index $c \in [\mathcal{N}]$, the probability of them

meeting is given by

$$P\left(c, O_1, \ldots, O_{\mathcal{N}}\right) = \underbrace{\left(\prod_{k \in I^{-1}(c)} N_k\right)^{-1}}_{\text{Probability of meeting in same cell}} \cdot \underbrace{\left(\sum_{a \in S(c)} O_a\right)}_{\text{Number of cells for meeting}}$$

when applying Assumptions 1 (uniform space use) and 3 (independence of movements).

Now we quantify the amount of unique information known by each subgroup in a way that aligns with Assumption 4. We consider the total number of cells in each of the core ranges, and then remove the number of cells known by all individuals, and ensure that all cells which is not known by all individuals are not over counted. To do this efficiently, we need to introduce more notation.

Let $f\colon [\mathcal{N}] \to \mathbb{R}$ be given by $f(c) = |I^{-1}(c)|$ by the number of individuals in the c-th grouping corresponding to the ordering given by I. Additionally, for brevity of notation, let $g\colon [\mathcal{N}] \to \mathbb{R}$ be given by $g(c) = f(c) - 1$. Define $B\colon [\mathcal{N}] \to \mathcal{P}([\mathcal{N}])$ such that $B(c)$ is the set of indices of all the smaller collections of individuals contained in the c-th collection of individuals:

$$B(c) = \left\{i \in [\mathcal{N}] : I^{-1}(i) \subset I^{-1}(c)\right\},$$

where $\subset$ is understood to mean *strict* containment. Then the amount of unique area known by the c-th subset of individuals can be written as

$$A\left(c, O_1, \ldots, O_{\mathcal{N}}\right) = \underbrace{\sum_{k \in I^{-1}(c)} N_k}_{\text{Sum of all areas}} - \underbrace{f(c) \sum_{a \in S(c)} O_a}_{\text{Area shared by all individuals}} - \underbrace{\sum_{a \in B(c)} g(a) O_a}_{\text{Areas shared by subgroups}}$$

where the second sum removes the number of cells which are known by all individuals in the group which are used for the transfer of information (which will be included $f(c)$ many times in the first sum) and the final sum ensures there is no over-counting of uniquely known cells (by removing all but one of the occasions upon which those cells were added to A). Then by Assumption 4, we may write the objective function as

$$\begin{aligned} T\left(O_1, \ldots, O_{\mathcal{N}}\right) &= \sum_{c=1}^{\mathcal{N}} P\left(c, O_1, \ldots, O_{\mathcal{N}}\right) A\left(c, O_1, \ldots, O_{\mathcal{N}}\right) \\ &= \sum_{c=1}^{\mathcal{N}} \left[\left(\frac{\sum_{a \in S(c)} O_a}{\prod_{k \in I^{-1}(c)} N_k}\right)\left(\sum_{k \in I^{-1}(c)} N_k - f(c) \sum_{a \in S(c)} O_a - \sum_{a \in B(c)} g(a) O_a\right)\right] \end{aligned}$$

which describes the net amount of information transfer. This is the sum of products of a linear function with an (affine) linear function, which implies that T is a quadratic form in $\mathcal{N}$ variables. Therefore, we can write T *uniquely* in the form

$$T(\boldsymbol{O}) = \boldsymbol{l}^t \boldsymbol{O} + \frac{1}{2} \boldsymbol{O}^t M \boldsymbol{O} \tag{2}$$

where $\boldsymbol{O} \in \mathbb{R}^{\mathcal{N}}$ is the vector of all the O_i's, $\boldsymbol{l} \in \mathbb{R}^{\mathcal{N}}$ and $M \in \mathbb{R}^{\mathcal{N} \times \mathcal{N}}$. We collect the terms in T to determine the vector $\boldsymbol{l}$ and matrix M. To do this, we first break the objective function into two parts, L and Q, which contain the linear and quadratic terms of T respectively, such that

$$\begin{aligned} T(\boldsymbol{O}) &= L(\boldsymbol{O}) - Q(\boldsymbol{O}), \\ L(\boldsymbol{O}) &= \sum_{c=1}^{\mathcal{N}} \left[\frac{\sum_{k \in I^{-1}(c)} N_k}{\prod_{k \in I^{-1}(c)} N_k} \sum_{a \in S(c)} O_a\right], \\ Q(\boldsymbol{O}) &= \sum_{c=1}^{\mathcal{N}} \left[\frac{1}{\prod_{k \in I^{-1}(c)} N_k}\left(f(c) \sum_{a_1, a_2 \in S(c)} O_{a_1} O_{a_2} + \sum_{\substack{a_1 \in B(c) \\ a_2 \in S(c)}} g(a_1) O_{a_1} O_{a_2}\right)\right]. \end{aligned}$$

We can determine the entries of $\boldsymbol{l}$ by finding the coefficients of the linear terms O_i in $T(\boldsymbol{O})$ for each $i \in [\mathcal{N}]$, which will be contained in $L(\boldsymbol{O})$. Let $i \in [\mathcal{N}]$. Then there is a contribution from the first sum (over c) to the coefficient of O_i only for $c \in [\mathcal{N}]$ such that $i \in S(c)$. For conciseness, we define $J\colon [\mathcal{N}] \to \mathcal{P}([\mathcal{N}])$ as

$$J(i) = \{c \in [\mathcal{N}] : i \in S(c)\}.$$

This allows us to write the coefficient of O_i in $L(\boldsymbol{O})$ as

$$\text{Coeff}(O_i) = \sum_{c \in J(i)} \left[\frac{\sum_{k \in I^{-1}(c)} N_k}{\prod_{k \in I^{-1}(c)} N_k} \right]$$

such that we can determine the entries of $\boldsymbol{l} = (l_i)$ as $l_i = \text{Coeff}(O_i)$.

We can also find the entries of the matrix Q by examining the coefficients of terms of the form $O_i O_j$ in $T(\boldsymbol{O})$ for all $i, j \in [\mathcal{N}]$, which will be contained in the $Q(\boldsymbol{O})$ part of $T(\boldsymbol{O})$. To simplify our argument, we break $Q(\boldsymbol{O})$ into two parts

$$\begin{aligned}
Q(\boldsymbol{O}) &= Q_1(\boldsymbol{O}) + Q_2(\boldsymbol{O}), \\
Q_1(\boldsymbol{O}) &= \sum_{c=1}^{\mathcal{N}} \left[\frac{1}{\prod_{k \in I^{-1}(c)} N_k} \left(f(c) \sum_{a_1, a_2 \in S(c)} O_{a_1} O_{a_2} \right) \right], \\
Q_2(\boldsymbol{O}) &= \sum_{c=1}^{\mathcal{N}} \left[\frac{1}{\prod_{k \in I^{-1}(c)} N_k} \left(\sum_{\substack{a_1 \in B(c) \\ a_2 \in S(c)}} g(a_1) O_{a_1} O_{a_2} \right) \right].
\end{aligned}$$

Let $i, j \in [\mathcal{N}]$. We first consider contributions to coefficients from Q_1. Here there is a contribution to $O_i O_j$ only for the values of c with $i \in S(c)$ and $j \in S(c)$, meaning the c values such that $I^{-1}(c) \subseteq I^{-1}(i)$ and $I^{-1}(c) \subseteq I^{-1}(j)$. These two conditions are equivalent to the single condition $I^{-1}(c) \subseteq I^{-1}(i) \cap I^{-1}(j)$. This now motivates the definition of a new function $V\colon [\mathcal{N}] \times [\mathcal{N}] \to [\mathcal{N}]$ with

$$V(i, j) = \left\{c : I^{-1}(c) \subseteq I^{-1}(i) \cap I^{-1}(j)\right\}.$$

For each c in this set, we will get a contribution of $2f(c)$ to the coefficient of $O_i O_j$ in Q_1 if $i \neq j$ (the scaling of 2 comes from the fact that the *unordered* pair (i, j) appears twice in the sum, from the cases $a_1 = i$, $a_2 = j$ and $a_1 = j$, $a_2 = i$) and a contribution of $f(c)$ if $i = j$.

This allows us to neatly write the the coefficient of $O_i O_j$ in T from Q_1, denoted $\text{Coeff}_1(O_i O_j)$, as

$$\text{Coeff}_1(O_i O_j) = \begin{cases} \sum_{c \in V(i,j)} \frac{2f(c)}{\prod_{k \in I^{-1}(c)} N_k}, & \text{if } i \neq j \\ \sum_{c \in V(i,j)} \frac{f(c)}{\prod_{k \in I^{-1}(c)} N_k}, & \text{if } i = j \end{cases}$$

Now we consider the contributions from Q_2. The c values for which the term $O_i O_j$ appears are those such that $i \in S(c)$ and $j \in B(c)$ (or the other way around, which is handled analogously). This is equivalent to the relevant c values being those such that $I^{-1}(c) \subseteq I^{-1}(i)$ and $I^{-1}(j) \subset I^{-1}(c)$. Note that this condition implies, by transitivity, that $I^{-1}(j) \subset I^{-1}(i)$, such that if this condition (or the reverse) is not satisfied, the contribution from this sum will be zero. That this implies all terms $O_i O_j$ with $i = j$ will not appear in Q_2. To collect all c values satisfying these two conditions, we define $Z\colon [\mathcal{N}] \times [\mathcal{N}] \to [\mathcal{N}]$ such that

$$Z(i : j) = \left\{c : I^{-1}(j) \subset I^{-1}(c) \subseteq I^{-1}(i)\right\}$$

where the colon notation is used to emphasise that $Z(i : j) \neq Z(j : i)$. This construction allows us to write the contribution from Q_2 as

$$\text{Coeff}_2(O_i O_j) = \begin{cases} \sum_{c \in Z(i:j)} \frac{g(j)}{\prod_{k \in I^{-1}(c)} N_k}, & \text{if } I^{-1}(j) \subset I^{-1}(i), \\ \sum_{c \in Z(j:i)} \frac{g(i)}{\prod_{k \in I^{-1}(c)} N_k}, & \text{if } I^{-1}(i) \subset I^{-1}(j), \\ 0, & \text{otherwise} \end{cases}$$

where $\text{Coeff}_2(O_i O_j)$ is the contribution of Q_2 to the coefficient of $O_i O_j$ in T. Finally, we add the contributions from Q_1 and Q_2 to determine the final coefficients of the second order terms in T as

$\text{Coeff}(O_iO_j) = \text{Coeff}_1(O_iO_j) + \text{Coeff}_2(O_iO_j)$. This collection of coefficients allows us to 'neatly' give the entries of the matrix $M = (M_{ij})$ as

$$M_{ij} = \begin{cases} -2\,\text{Coeff}(O_iO_j), \text{ if } i = j \\ -\text{Coeff}(O_iO_j), \text{ otherwise} \end{cases}$$

where the sign is reversed because $Q(\boldsymbol{O})$ has a coefficient of -1 in $T(\boldsymbol{O})$. Note, therefore, that all entries of M_{ij} will be non-positive. We note here for the next section, that since we double the diagonal terms (where $i = j$), then the effective contribution from $\text{coeff}_1(O_iO_j)$ to M is always

$$m_{ij} = -\sum_{c \in V(i,j)} \frac{2f(c)}{\prod_{k \in I^{-1}(c)} N_k}$$

We now have the objective form written in the standard quadratic form $T(\boldsymbol{O}) = \boldsymbol{l}^t\boldsymbol{O} + \frac{1}{2}\boldsymbol{O}^t M\boldsymbol{O}$. We now need to impose some constraints on the problem to ensure that the solution makes biological sense. In particular,

- All areas should be non-negative.
- The sum of all cells in which an individual has shared knowledge of is less than their total area.

Mathematically these conditions can be written as

- $\boldsymbol{O} \geq 0$, meaning that $O_i \geq 0$, $\forall i \in [\mathcal{N}]$.
- $\sum_{i \in S(I(\{k\}))} O_i \leq N_k$, $\forall k \in [n]$.

The second condition (no over-sharing) is best represented in the form $\boldsymbol{G}\boldsymbol{O} \leq \boldsymbol{h}$ for some matrix $G \in \mathbb{R}^{n \times \mathcal{N}}$ and some vector $h \in \mathbb{R}^n$. Thankfully, these are easier to construct than the previous matrix and vector. The vector $\boldsymbol{h} = (h_i)$ will have entries $h_i = N_i$, and the matrix $G = (G_{ij})$ will have entries

$$G_{ij} = \begin{cases} 1, \text{ if } j \in S(I(\{i\})) \\ 0, \text{ otherwise} \end{cases}$$

which deals with our conditions. Finally, we can write down the optimisation problem as

$$\begin{aligned} \max_{\boldsymbol{O}} \quad & \boldsymbol{l}^t\boldsymbol{O} + \frac{1}{2}\boldsymbol{O}^t M\boldsymbol{O} \\ \text{s.t.} \quad & \boldsymbol{G}\boldsymbol{O} \leq \boldsymbol{h} \\ & \boldsymbol{O} \geq 0 \end{aligned}$$

Optimiser in the homogeneous case

Suppose that the population is homogeneous in foraging ability. Meaning, $N_k = N > 0$ for $k \in [\mathcal{N}]$.

Proposition 1. Suppose $N_k = N > 0$ $\forall k \in [\mathcal{N}]$. Let $\hat{f}\colon [\mathcal{N}] \times [\mathcal{N}] \to \mathbb{N}$ be defined by

$$\hat{f}(i,j) = |I^{-1}(i) \cap I^{-1}(j)|$$

Then all of the coefficients in the objective function $T(\mathbf{O})$ can be expressed as

$$l_i = N\sum_{k=2}^{f(i)} \binom{f(i)}{k} kN^{-k},$$

$$M_{ij} = m_{ij,1} + m_{ij,2}, \text{where:}$$

$$m_{ij,1} = \begin{cases} -2\sum_{k=2}^{\hat{f}(i,j)} \binom{\hat{f}(i,j)}{k} kN^{-k}, \text{ if } \hat{f}(i,j) \geq 2 \\ 0, \text{ otherwise} \end{cases},$$

$$m_{ij,2} = \begin{cases} -\sum_{k=1}^{\hat{f}(i,j)} \binom{\hat{f}(i,j)}{k} (f(j) + k - 1)N^{-f(i)-k}, \text{ if } I^{-1}(j) \subset I^{-1}(i) \\ 0, \text{ otherwise} \end{cases}$$

$$M_{ji} = M_{ij}$$

where $i, j \in [\mathcal{N}]$ and $i \geq j$.

Proof. First we deal with the linear terms given in the vector $\boldsymbol{l} = (l_i)$. In generality, these are given by

$$l_i = \sum_{c \in J(i)} \left[\frac{\sum_{k \in I^{-1}(c)} N_k}{\prod_{k \in I^{-1}(c)} N_k} \right]$$

which under the homogeneity assumption (that $N_k = N \ \forall c \in [\mathcal{N}]$), this simplifies to:

$$l_i = \sum_{c \in J(i)} \left[\frac{f(c)N}{N^{f(c)}} \right] = \sum_{c \in J(i)} f(c) N^{1-f(c)}.$$

The set $J(i)$ is the collection of indices $c \in [\mathcal{N}]$ such that the set $I^{-1}(c) \subseteq I^{-1}(i)$. Since $f(i)$ is the size of the set $I^{-1}(i)$, there are $\binom{f(i)}{k}$ many subsets of $I^{-1}(i)$ of size k for $k = 2 \ldots, f(i)$. Since l_i depends only upon the size of the subsets under the homogeneity assumption, we can group terms in the sum by their size. Doing this, we obtain the required expression

$$l_i = \sum_{k=2}^{f(i)} k N^{1-k} = N \sum_{k=2}^{f(i)} k N^{-k}.$$

We first compute $m_{ij,1}$. This has the form:

$$m_{ij,1} = - \sum_{c \in V(i,j)} \frac{2f(c)}{\prod_{k \in I^{-1}(c)} N_k} = -2 \sum_{c \in V(i,j)} f(c) N^{-f(c)}$$

The set $V(i,i)$, by definition, is given by

$$V(i,j) = \left\{ c : I^{-1}(c) \subseteq I^{-1}(i) \cap I^{-1}(j) \right\}.$$

The size of the set $I^{-1}(i) \cap I^{-1}(j)$ is given by $\hat{f}(i,j)$ by definition. If $\hat{f}(i,j) = 0$ or $\hat{f}(i,j) = 1$, then $V(i,j)$ should be empty since we do not consider subgroups of size 0 or 1. If $\hat{f}(i,j) \geq 2$, then we can group terms in the sum by their corresponding subgroup size again, as done with the linear coefficients:

$$m_{ij,1} = -2 \sum_{k=2}^{\hat{f}(i,j)} \binom{\hat{f}(i,j)}{k} k N^{-k}.$$

Now we focus on $m_{ij,2}$. In the previous subsection, this is denoted by $-\operatorname{Coeff}_2(O_i O_j)$. Since $i \geq j$, we cannot have that $I^{-1}(i) \subset I^{-1}(j)$ by the definition of the index map I. If $I^{-1}(j) \not\subset I^{-1}(i)$ then $m_{ij,2} = 0$. If $I^{-1}(j) \subset I^{-1}(i)$, then

$$m_{ij,2} = - \sum_{c \in Z(i:j)} \frac{g(j)}{\prod_{k \in I^{-1}(c)} N_k} = - \sum_{c \in Z(i:j)} g(j) N^{-f(c)}.$$

The set $Z(i:j)$, by definition, is given by

$$Z(i:j) = \{ c : I^{-1}(j) \subset I^{-1}(c) \subseteq I^{-1}(i) \}$$

and therefore gives the number of 'intermediate' sets between $I^{-1}(j)$ and $I^{-1}(i)$, including $I^{-1}(i)$ but excluding $I^{-1}(j)$. We therefore can break subsets again into their size, by noting that the number of intermediate sets of size will contain $f(j)$ and some additional elements from $f(i)$ not already contained in $f(j)$. The number of sets with k additional elements will be $\binom{f(i)-f(j)}{k}$ for $k = 1, \ldots, f(i) - f(j)$. These sets will be of size $f(j) + k$. Therefore, in the case of $I^{-1}(j) \subset I^{-1}(i)$, we can write:

$$m_{ij,2} = - \sum_{k=1}^{f(i)-f(j)} \binom{f(i) - f(j)}{k} (f(j) + k - 1) N^{-f(j)-k}.$$

Then observing that $\hat{f}(i,j) = f(i) - f(j)$ under the condition $f(i) - f(j)$, we can express

$$m_{ij,2} = - \sum_{k=1}^{\hat{f}(i,j)} \binom{\hat{f}(i,j)}{k} (f(j) + k - 1) N^{-f(j)-k}.$$

Then $M_{ij} = m_{ij,1} + m_{ij,2}$ for $i \geq j$, giving all of the lower-diagonal entries. By the symmetry of M we have all coefficients of T simplified using the homogeneity assumption.

□

Definition. The *feasible region* is the set

$$\Omega = \{\boldsymbol{O} \in \mathbb{R}^{\mathcal{N}} : G\boldsymbol{O} \leq \boldsymbol{h}, \boldsymbol{O} \geq 0\}.$$

Meaning, the set of points in which the constraints of the problem (as given in the previous section) are not violated.

Definition. The *feasible perturbation region about a point* $\boldsymbol{O} \in \mathbb{R}^{\mathcal{N}}$ is the set

$$\Omega_\varepsilon(\boldsymbol{O}) = \{\boldsymbol{\varepsilon} \in \mathbb{R}^{\mathcal{N}} : \boldsymbol{O} + \boldsymbol{\varepsilon} \in \Omega\}$$

Meaning, the set of perturbations from the point $\boldsymbol{O}$ which remain in the feasible set.

Lemma 1. A feasible point $O^* \in \Omega$ is a local maximiser of T if and only if there exists a neighbourhood $U \in \Omega_\varepsilon(O^*)$ around $\mathbf{0}$ such that every $\varepsilon \in U$ satisfies

$$T(\varepsilon) \leq -O^* M\varepsilon. \tag{3}$$

Furthermore, if this property holds for $U = \Omega_\varepsilon(O^*)$, then O^* is a global maximiser of T. We refer to this inequality as the optimality condition for O^* with perturbation ε.

Proof. The condition for a feasible point $O^* \in \Omega$ to be a local maximiser of T (*the optimality condition*) is that there exists some $\epsilon > 0$ such that $T(O) \leq T(O^*)$ for all $O \in \Omega$ satisfying $\|O - O^*\| \leq \epsilon$.

Any $O \in \Omega$ can be written as $O = O^* + \varepsilon$ for some $\varepsilon \in \Omega_\varepsilon(O^*)$, since both O and O^* are in Ω and by the definition of $\Omega_\varepsilon(O^*)$. Using this expression for O, we can write $\|O - O^*\| = \|\varepsilon\|$. Therefore, the optimality condition can be rewritten as follows. A feasible point $O^* \in \Omega$ is a local maximiser of T if there exists some $\epsilon > 0$ such that $T(O^* + \varepsilon) \leq T(O^*)$ for all $\varepsilon \in \Omega_\varepsilon(O^*)$ satisfying $\|\varepsilon\| \leq \epsilon$. This is equivalent to the point O^* being optimal if there exists some neighbourhood $U \in \Omega_\varepsilon(O^*)$ around $\mathbf{0}$ such that every $\varepsilon \in U$ satisfies $T(O^* + \varepsilon) \leq T(O^*)$. Then observe that

$$\begin{aligned}
T(O^* + \varepsilon) &= \frac{1}{2}(O^{*t} + \varepsilon^t)M(O^* + \varepsilon) + l^t(O^* + \varepsilon) \\
&= \frac{1}{2}O^{*t}MO^* + \frac{1}{2}O^{*t}M\varepsilon + \frac{1}{2}\varepsilon^t MO^* + \frac{1}{2}\varepsilon^t M\varepsilon + l^t O^* + l^t\varepsilon \\
&= T(O^*) + T(\varepsilon) + \frac{1}{2}O^{*t}M\varepsilon + \frac{1}{2}\varepsilon^t MO^* \\
&= T(O^*) + T(\varepsilon) + O^{*t}M\varepsilon, \text{ since } M \text{ is symmetric, so } O^{*t}M\varepsilon = \varepsilon^t MO^*.
\end{aligned}$$

This implies the equivalence

$$\begin{aligned}
T(O^* + \varepsilon) \leq T(O^*) &\iff T(O^* + \varepsilon) - T(O^*) \leq 0 \\
&\iff T(\varepsilon) + O^{*t}M\varepsilon \leq 0 \\
&\iff T(\varepsilon) \leq -O^{*t}M\varepsilon.
\end{aligned}$$

Therefore, the optimality condition is equivalent to the statement that there exists some neighbourhood $U \in \Omega_\varepsilon(O^*)$ around $\mathbf{0}$ such that every $\varepsilon \in U$ satisfies:

$$T(\varepsilon) \leq -O^{*t}M\varepsilon.$$

Replacing U with $\Omega_\varepsilon(O^*)$ throughout analogously gives the condition for global optimality. □

Lemma 2. Let $O^* = (0, \ldots, 0, \frac{N}{2})$ and $\varepsilon = (\varepsilon_1, \ldots, \varepsilon_{\mathcal{N}}) \in \Omega_{O^*}$ be such that $\varepsilon_i \geq 0 \ \forall i \in [\mathcal{N}]$. Then ε satisfies $T(\varepsilon) \leq -O^{*t}M\varepsilon$.

Proof. Let $\varepsilon = (\varepsilon_i) \in \Omega_\varepsilon(O^*)$ be any perturbation of the allowed form, where $\varepsilon_i \geq 0$ for $i =$

$1, \dots, \mathcal{N}$. Define $v = (v_i) = O^{*t}M$. Then observe

$$\begin{aligned} v_i &= \sum_{j=1}^{\mathcal{N}} M_{ji} O_j \\ &= M_{\mathcal{N}i} \frac{N}{2} \\ &= \left(-2 \sum_{k=2}^{f(i)} \binom{f(i)}{k} k N^{-k} - \sum_{k=1}^{n-f(i)} \binom{n-f(i)}{k} (f(i)+k-1) N^{-f(i)-k} \right) \frac{N}{2} \\ &= -N \sum_{k=2}^{f(i)} \binom{f(i)}{k} k N^{-k} - \frac{N}{2} \sum_{k=1}^{n-f(i)} \binom{n-f(i)}{k} (f(i)+k-1) N^{-f(i)-k} \\ &= -l_i - \frac{N}{2} \sum_{k=1}^{n-f(i)} \binom{n-f(i)}{k} (f(i)+k-1) N^{-f(i)-k}. \end{aligned}$$

Therefore, $v_i \leq -l_i$, since the second term in the right hand side of the final expression is strictly negative. This implies that

$$\sum_{i=1}^{\mathcal{N}} l_i \varepsilon_i \leq -\sum_{i=1}^{\mathcal{N}} v_i \varepsilon_i.$$

Therefore, since all of the elements of M are non-positive, we also have that

$$\frac{1}{2} \sum_{i=1}^{\mathcal{N}} \sum_{j=1}^{\mathcal{N}} \varepsilon_i \varepsilon_j M_{ij} + \sum_{i=1}^{\mathcal{N}} l_i \varepsilon_i \leq -\sum_{i=1}^{\mathcal{N}} v_i \varepsilon_i.$$

Which is equivalent to

$$T(\varepsilon) \leq -v^t \varepsilon = -O^{*t} M \varepsilon$$

as required.

□

Lemma 3. Let $O^* = (0, \dots, 0, \frac{N}{2})$ and $\varepsilon = (0, \dots, 0, -\varepsilon_{\mathcal{N}})$ be such that $\varepsilon_{\mathcal{N}} > 0$. Then ε satisfies $T(\varepsilon) \leq -O^{*t} M \varepsilon$.

Proof. Let $\varepsilon = (\varepsilon_i) \in \Omega_\varepsilon(O^*)$ be any perturbation of the allowed form, where $\varepsilon_i = 0$ for $i = 1, \dots, \mathcal{N}-1$ and $\varepsilon_{\mathcal{N}} < 0$. Observe the following equivalence:

$$\begin{aligned} T(\varepsilon) \leq -O^{*t} M \varepsilon &\iff \frac{1}{2} \varepsilon^t M \varepsilon + l^t \varepsilon \leq -O^{*t} M \varepsilon \\ &\iff \sum_{i=1}^{\mathcal{N}} \sum_{j=1}^{\mathcal{N}} \varepsilon_i M_{ij} \varepsilon_j + \sum_{i=1}^{\mathcal{N}} \varepsilon_i l_i \leq -\sum_{i=1}^{\mathcal{N}} \sum_{j=1}^{\mathcal{N}} O_i^* M_{ij} \varepsilon_j \\ &\iff \varepsilon_{\mathcal{N}}^2 M_{\mathcal{N}\mathcal{N}} + \varepsilon_{\mathcal{N}} l_{\mathcal{N}} \leq -O_{\mathcal{N}}^* M_{\mathcal{N}\mathcal{N}} \varepsilon_{\mathcal{N}} \\ &\iff \varepsilon_{\mathcal{N}} M_{\mathcal{N}\mathcal{N}} + l_{\mathcal{N}} \geq -O_{\mathcal{N}}^* M_{\mathcal{N}\mathcal{N}} \\ &\iff (\varepsilon_{\mathcal{N}} + O^*) M_{\mathcal{N}\mathcal{N}} + l_{\mathcal{N}} \geq 0 \\ &\iff -2 \left(\varepsilon_{\mathcal{N}} + \frac{N}{2} \right) \left(\sum_{k=2}^{n} \binom{n}{k} k N^{-k} \right) + N \left(\sum_{k=2}^{n} \binom{n}{k} k N^{-k} \right) \geq 0 \\ &\iff -2\varepsilon_{\mathcal{N}} - N + N \geq 0 \\ &\iff \varepsilon_{\mathcal{N}} \leq 0 \end{aligned}$$

which is true by assumption. □

Remark. Any $\varepsilon \in \Omega_{O^*}$ can be written in the form of $\varepsilon^{(1)}$, as defined in Lemma 2, or in the form of $\varepsilon^{(1)} + \varepsilon^{(2)}$, where $\varepsilon^{(2)}$ is as defined in Lemma 3. These two cases correspond to the distinction between feasible perturbations in the positive and negative directions with respect to the final coordinate $O_{\mathcal{N}}$, respectively. Other coordinates must be perturbed from O^* in the positive direction, since O^* lies on the boundary of Ω (as $O_i = 0$ for $i = 1, \dots, \mathcal{N}-1$).

Theorem 1. Let $O^* = (0, \ldots, 0, \frac{N}{2})$. Then O^* is the global maximiser of T.

Proof. We construct a neighbourhood U about $\mathbf{0}$ in $\Omega_\varepsilon(O^*)$ such that all $\varepsilon \in U$ satisfy

$$T(\varepsilon) \leq -O^{*t} M\varepsilon \tag{4}$$

which would imply that O^* satisfies the optimality condition of Lemma 1 in the local sense. We then show that this constructed set must be equal to $\Omega_\varepsilon(O^*)$ itself, so that O^* also satisfies the optimality condition of Lemma 1 in the global sense. To construct this set, we consider which perturbations in $\Omega_\varepsilon(O^*)$ satisfy inequality (4). As noted in the previous remark, each feasible perturbation can be written in the form of $\varepsilon^{(1)}$, or in the form of $\varepsilon^{(1)} + \varepsilon^{(2)}$. We consider these two cases separately.

In the first case, all perturbations are of the form $\varepsilon = (\varepsilon_i)$ and $\varepsilon_i \geq 0$. By Lemma 2, the condition (4) holds for any such $\varepsilon \in \Omega_\varepsilon(O^*)$.

In the second case, perturbations are of the form $\varepsilon = \varepsilon^{(1)} + \varepsilon^{(2)}$. Denote $\varepsilon^{(1)} = (\varepsilon_i^{(1)})$ and $\varepsilon^{(2)} = (\varepsilon_i^{(2)})$. Perturbations of this form will have $\varepsilon_i^{(1)} \geq 0$ for $i = 1, \ldots, \mathcal{N}$, $\varepsilon_j^{(2)} \geq 0$ for $i = 1, \ldots, \mathcal{N} - 1$ and $\varepsilon_{\mathcal{N}}^{(2)} < 0$. Without loss of generality, assume that $\varepsilon_{\mathcal{N}}^{(1)} = 0$. Now observe that:

$$\begin{aligned}
T(\varepsilon) &= T\left(\varepsilon^{(1)} + \varepsilon^{(2)}\right) \\
&= \frac{1}{2}\left(\varepsilon^{(1)} + \varepsilon^{(2)}\right)^t M\left(\varepsilon^{(1)} + \varepsilon^{(2)}\right) + l^t\left(\varepsilon^{(1)} + \varepsilon^{(2)}\right) \\
&= T\left(\varepsilon^{(1)}\right) + T\left(\varepsilon^{(2)}\right) + \varepsilon^{(1)t} M\varepsilon^{(2)} \\
&\leq T(\varepsilon^{(1)}) - O^{*t} M\varepsilon^{(2)} + \varepsilon^{(1)t} M\varepsilon^{(2)}, \text{ by Lemma 3.}
\end{aligned}$$

Furthermore, by Lemma 2, we have that

$$T\left(\varepsilon^{(1)}\right) \leq -O^* M\varepsilon^{(1)},$$

which implies that

$$X := -T\left(\varepsilon^{(1)}\right) - O^* M\varepsilon^{(1)} \tag{5}$$

is non-negative. Our current inequality for $T(\varepsilon)$ can be expressed as

$$T(\varepsilon) \leq -O^{*t} M\varepsilon^{(1)} - X - O^{*t} M\varepsilon^{(2)} + \varepsilon^{(1)t} M\varepsilon^{(2)},$$

which implies that

$$T(\varepsilon) \leq -O^{*t} M\varepsilon + \left(-X + \varepsilon^{(1)t} M\varepsilon^{(2)}\right).$$

Therefore, ε will satisfy the optimality condition (4) if

$$-X + \varepsilon^{(1)t} M\varepsilon^{(2)} \leq 0 \iff X \geq \varepsilon^{(1)t} M\varepsilon^{(2)}.$$

Expanding the $\varepsilon^{(1)t} M\varepsilon^{(2)}$ term gives:

$$\varepsilon^{(1)t} M\varepsilon^{(2)} = \sum_{i=1}^{\mathcal{N}} \sum_{j=1}^{\mathcal{N}} \varepsilon_j^{(1)} M_{ij} \varepsilon_i^{(2)} = \sum_{j=1}^{\mathcal{N}} \varepsilon_j^{(1)} M_{\mathcal{N}j} \varepsilon_{\mathcal{N}} = \varepsilon_{\mathcal{N}} \sum_{j=1}^{\mathcal{N}} \varepsilon_j^{(1)} M_{\mathcal{N}j}.$$

Therefore, ε will satisfy the optimality condition (4) if

$$X \geq \varepsilon_{\mathcal{N}} \sum_{j=1}^{\mathcal{N}} \varepsilon_j^{(1)} M_{\mathcal{N}j} \implies \varepsilon_{\mathcal{N}} \geq \frac{X}{\sum_{j=1}^{\mathcal{N}} \varepsilon_j^{(1)} M_{\mathcal{N}j}},$$

using the fact that all entries of M are negative and all elements of $\varepsilon^{(1)}$ are positive, which implies the above sum is negative. We use this property to construct the set U.

Consider the map $P\colon \Omega_\varepsilon(O^*) \to \mathbb{R}^{\mathcal{N}}$ which maps the final coordinate to zero and fixes the remaining coordinates. Define $U' = P(\Omega_\varepsilon(O^*))$ and

$$K := \min_{\varepsilon \in U'} \left[\frac{X}{\sum_{j=1}^{\mathcal{N}} \varepsilon_j^{(1)} M_{\mathcal{N}j}}\right],$$

which must exist, be finite and be non-zero since $N \neq 0$. Using the definition of X we can write

$$\begin{aligned} K &= \min_{\varepsilon \in U'} \left[\frac{-T\left(\varepsilon^{(1)}\right) - O^* M \varepsilon^{(1)}}{\sum_{j=1}^{\mathcal{N}} \varepsilon_j^{(1)} M_{\mathcal{N}j}} \right] \\ &= \min_{\varepsilon \in U'} \left[\frac{-T\left(\varepsilon^{(1)}\right) - \frac{N}{2} \sum_{j=1}^{\mathcal{N}} \varepsilon_j^{(1)} M_{\mathcal{N}j}}{\sum_{j=1}^{\mathcal{N}} \varepsilon_j^{(1)} M_{\mathcal{N}j}} \right] \\ &= \min_{\varepsilon \in U'} \left[\frac{-T\left(\varepsilon^{(1)}\right)}{\sum_{j=1}^{\mathcal{N}} \varepsilon_j^{(1)} M_{\mathcal{N}j}} \right] - \frac{N}{2} \end{aligned}$$

Now observe that the quantity in the minimisation operator can take the value 0, exactly when $T(\varepsilon) = 0$. We can construct a value of $\varepsilon \in U' \setminus \{0\}$ which solves $T(\varepsilon)$ as follows. Split $[n]$ into two *disjoint* subsets, S_1 and S_2, consisting of $\lceil \frac{n}{2} \rceil$ and $\lfloor \frac{n}{2} \rfloor$ many individuals, respectively. Set $\varepsilon_j = N$ for $j = I(S_1)$ and $j = I(S_2)$, and all other coordinate values $\varepsilon_i = 0$. Therefore, for all $c \in [\mathcal{N}]$, we have that either $P(c) = 0$ (for the zero overlaps) or $A(c) = 0$ (for the two non-zero overlaps of individuals, where the amount of unique knowledge is zero), which implies that the net information transfer, $T(\varepsilon)$, is 0. Therefore, we can say

$$K \leq -\frac{N}{2}$$

Then, we can define $U_{\mathcal{N}}$ as

$$U_{\mathcal{N}} = \{0\} \times \cdots \times \{0\} \times \left[-\frac{N}{2}, \frac{N}{2}\right]$$

and then define U as

$$U = (U' + U_{\mathcal{N}}) \cap \Omega_\varepsilon(O^*)$$

where the + denotes the Minkowski sum of sets (i.e the element-wise sum). By construction, U is a neighbourhood in $\Omega_\varepsilon(O^*)$ about $\mathbf{0}$ where the condition

$$T(\varepsilon) \leq -O^{*t} M \varepsilon \tag{6}$$

holds for all $\varepsilon \in U$. Therefore, by Lemma 1, O^* is a local optimiser of T. For global optimality, it remains to show that $U = \Omega_\varepsilon(O^*)$. We do this by showing that

$$\Omega_\varepsilon(O^*) \subseteq U' + U_{\mathcal{N}}$$

which would imply that $U = \Omega_\varepsilon(O^*)$. Let ε be any feasible perturbation. We show that this implies $\varepsilon \in U' + U_{\mathcal{N}}$. Write $\varepsilon = \varepsilon^{(1)} + \varepsilon^{(2)}$, where $\varepsilon^{(1)}$ contains the first $\mathcal{N} - 1$ entries of ε but has $\varepsilon_{\mathcal{N}}^{(1)} = 0$, and $\varepsilon^{(2)}$ has $\varepsilon_i^{(2)} = 0$ for all $i \in [\mathcal{N}]$ except for $\mathcal{N}$. We therefore note that, by definition, $\varepsilon^{(1)} = P(\varepsilon)$, so that $\varepsilon^{(1)}$ is in the image $P(\Omega_\varepsilon(O^*))$. We show that $\varepsilon^{(2)}$ must be in $U_{\mathcal{N}}$. This is a straightforward argument. If $\varepsilon_{\mathcal{N}} < -\frac{N}{2}$, then $O^* + \varepsilon$ does not satisfy the constraint $\boldsymbol{O} \geq 0$. So, no $\varepsilon \in \Omega_\varepsilon(O^*)$ can have this property. So, on the other hand, if $\varepsilon_{\mathcal{N}} > \frac{N}{2}$, then the condition $G(O^* + \varepsilon) \leq h$ can not be satisfied since

$$(O^* + \varepsilon)_{\mathcal{N}} = \frac{N}{2} + \varepsilon_{\mathcal{N}} > N,$$

such that no $\varepsilon \in \Omega_\varepsilon(O^*)$ can have this property either. Hence, $\varepsilon^{(2)} \in U_{\mathcal{N}}$. Therefore, $\varepsilon \in U' + U_{\mathcal{N}}$. This implies that

$$U = (U' + U_{\mathcal{N}}) \cap \Omega_\varepsilon(O^*) = \Omega_\varepsilon(O^*)$$

which therefore proves that O^* is the global optimiser of T over Ω. □

Remark. At the global optimiser $O^* = (0, \ldots, 0, \frac{N}{2})$, the objective function takes value

$$T(O^*) = \frac{N^2}{2} \sum_{k=2}^{n} \binom{n}{k} k N^{-k} = \frac{n(n-1)}{4} + \mathcal{O}(N^{-1})$$

and therefore, when the 'number of points' is very large, the optimal value of the information transfer function is approximately quadratic in the number of individuals in the group.

Definition. The *total relative overlap of the group* of n individuals, w_n, is defined as the ratio of the number of cells known by *all* individuals to the number of cells known by at least one individual. In particular:

$$w_n = \frac{O_{\mathcal{N}}}{\sum_{i=1}^{n} N_i - \sum_{c=1}^{\mathcal{N}} g(c) O_c}$$

or, equivalently, the ratio of the area shared by all individuals to the total occupied area.

This definition is general to both the homogenous and non-homogenous case. But the following result holds only in the homogenous one.

Corollary. At the optimal rate of information transfer, the total relative overlap of the group of n individuals is given by:

$$w_n^* = \frac{1}{n+1}$$

Proof. By Theorem 1, the optimiser of T is $O^* = (0, \dots, 0, \frac{N}{2})$. The corresponding value of w_n is then given by

$$\begin{aligned} w_n^* &= \frac{O_{\mathcal{N}}}{\sum_{i=1}^{n} N_i - \sum_{c=1}^{\mathcal{N}} g(c) O_c} \\ &= \frac{N/2}{nN - g(\mathcal{N})N/2} \\ &= \frac{1}{2n - (n-1)}, \text{ cancelling the } N/2 \text{ term} \\ &= \frac{1}{n+1}. \end{aligned}$$

□

2 Results

2.1 Correlation between variation in fruit abundance and the filtration complementarity index

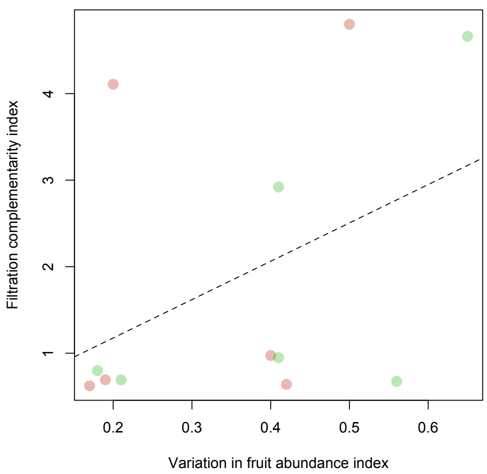

Figure 1: Relationship between the confidence intervals in the biweekly index of fruit abundance and the filtration complementarity index for different seasons. The values of the filtration complementarity index do not vary significantly with season, although they show a tendency in the predicted direction (ANOVA F_1=2.2, p =0.17). Light red: dry seasons; light green: wet seasons. The dotted line corresponds to the non-significant linear fit to all points, regardless of season (ρ = 0.42 and P=0.16).

2.2 Simplicial centrality and other simplex characteristics

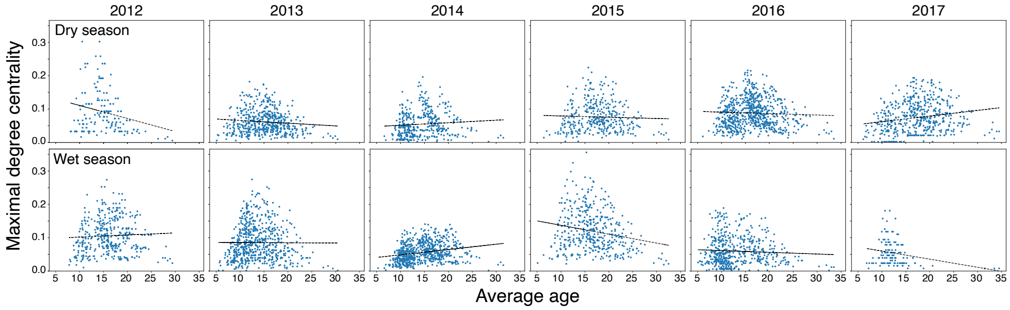

Figure 2: Maximal simplicial degree centrality values for simplices composed of adult individuals of different age. Each dot corresponds to a simplex formed by the intersection of individual core ranges assuming $\alpha \leq 4$. Top row: dry seasons; bottom row: wet seasons.

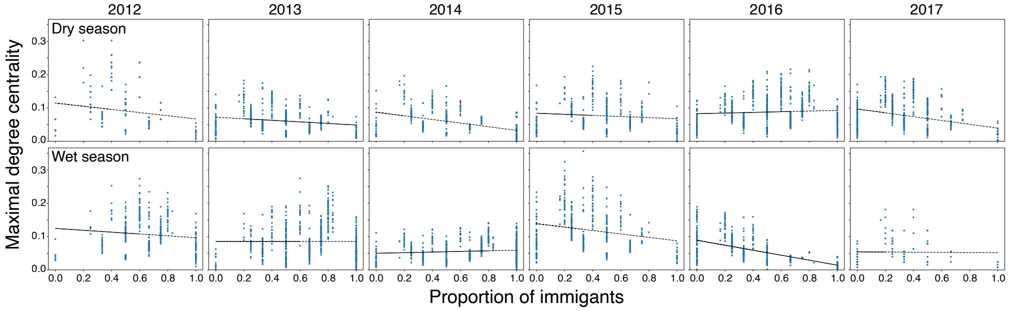

Figure 3: Maximal simplicial degree centrality values for simplices with different proportions of males. Each dot corresponds to a simplex formed by the intersection of individual core ranges assuming $\alpha \leq 4$. Top row: dry seasons; bottom row: wet seasons.

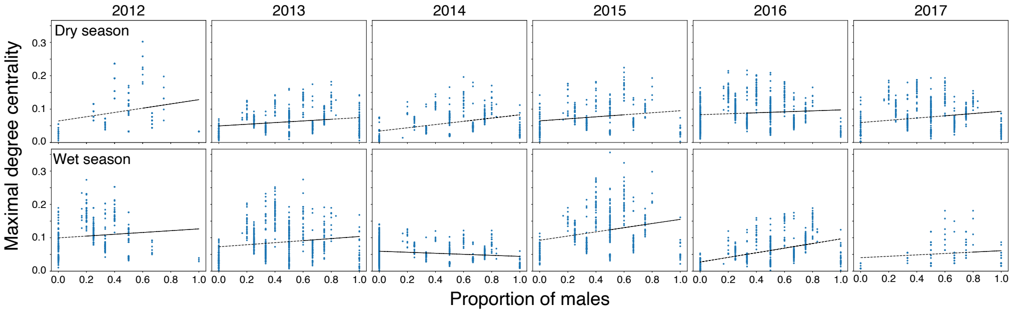

Figure 4: Maximal simplicial degree centrality values for simplices composed of different proportions of recently immigrated females. Each dot corresponds to a simplex formed by the intersection of individual core ranges assuming $\alpha \leq 4$. Top row: dry seasons; bottom row: wet seasons.

2.3 Simplicial centrality and maximum simplex size

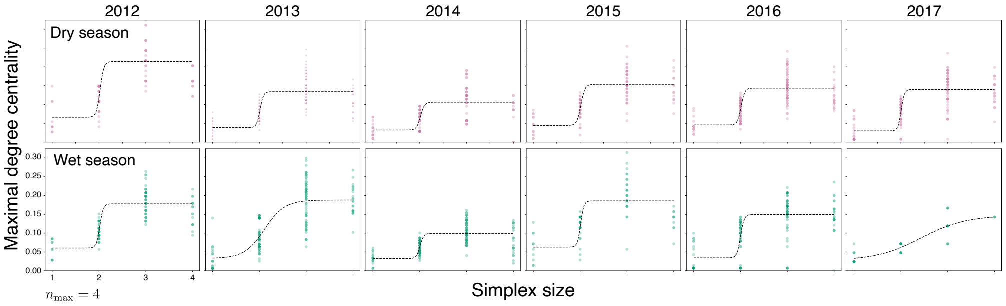

Figure 5: Maximal simplicial degree centrality values for simplices of different size with maximum simplex size parameter $n_{\max} = 4$. Each dot corresponds to a simplex formed by the intersection of individual core ranges assuming $\alpha \leq 4$. Dashed black line corresponds to a continuous sigmoid function fitted to the centrality values using the non-linear least squares method from the *Scipy* package in Python [1]. Top row: dry seasons; bottom row: wet seasons.

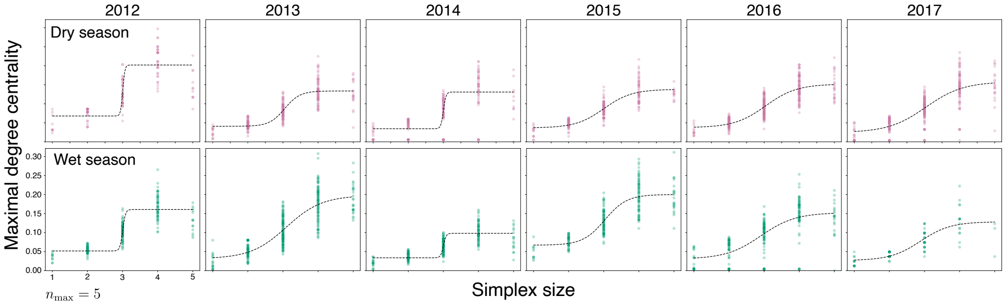

Figure 6: Maximal simplicial degree centrality values for simplices of different size with maximum simplex size parameter $n_{\max} = 5$. Each dot corresponds to a simplex formed by the intersection of individual core ranges assuming $\alpha \leq 4$. Dashed black line corresponds to a continuous sigmoid function fitted to the centrality values using the non-linear least squares method from the *Scipy* package in Python [1]. Top row: dry seasons; bottom row: wet seasons.

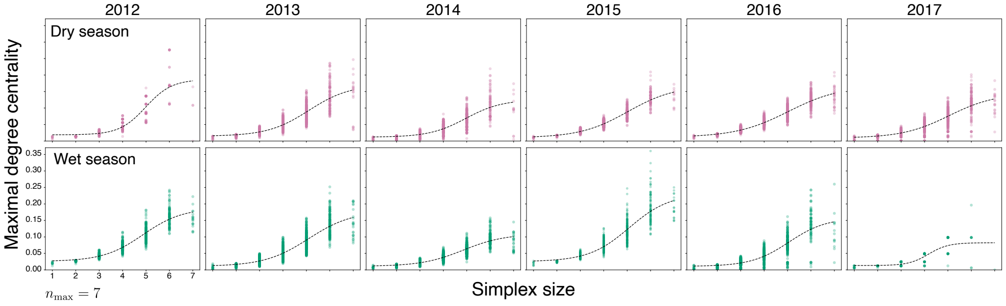

Figure 7: Maximal simplicial degree centrality values for simplices of different size with maximum simplex size parameter $n_{\max} = 7$. Each dot corresponds to a simplex formed by the intersection of individual core ranges assuming $\alpha \leq 4$. Dashed black line corresponds to a continuous sigmoid function fitted to the centrality values using the non-linear least squares method from the *Scipy* package in Python [1]. Top row: dry seasons; bottom row: wet seasons.

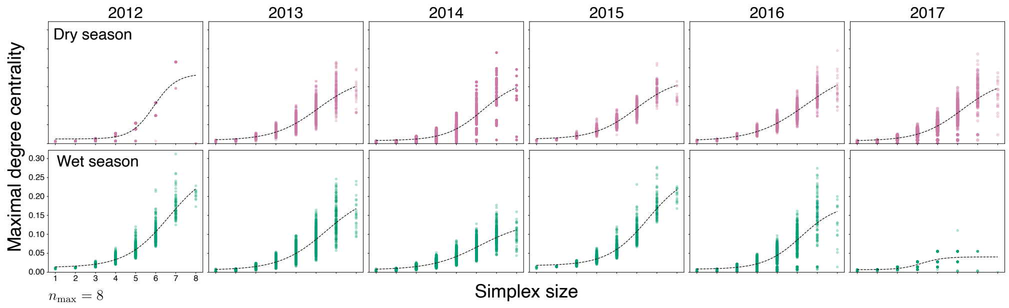

Figure 8: Maximal simplicial degree centrality values for simplices of different size with maximum simplex size parameter $n_{\max} = 8$. Each dot corresponds to a simplex formed by the intersection of individual core ranges assuming $\alpha \leq 4$. Dashed black line corresponds to a continuous sigmoid function fitted to the centrality values using the non-linear least squares method from the *Scipy* package in Python [1]. Top row: dry seasons; bottom row: wet seasons.

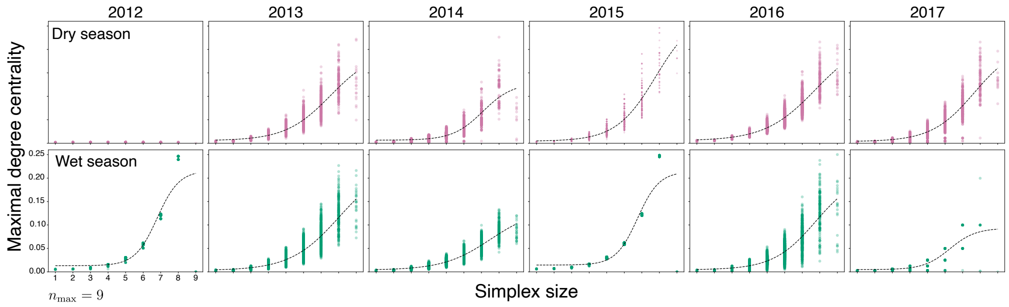

Figure 9: Maximal simplicial degree centrality values for simplices of different size with maximum simplex size parameter $n_{\max} = 9$. Each dot corresponds to a simplex formed by the intersection of individual core ranges assuming $\alpha \leq 4$. Dashed black line corresponds to a continuous sigmoid function fitted to the centrality values using the non-linear least squares method from the *Scipy* package in Python [1]. Top row: dry seasons; bottom row: wet seasons. No sigmoid was fitted for the dry season of 2012 due to low centrality values (which arise due to the trivial structure).
